# Efficacy of rituximab on disease activity, severity, and disease-related damage in patients with immunosuppressive-resistant systemic sclerosis

**DOI:** 10.55730/1300-0144.5739

**Published:** 2023-10-12

**Authors:** Yasemin YALÇINKAYA, Bahar ARTIM ESEN, Shirkhan AMİKİSHİYEV, Numuna ALİYEVA, Ahmet GÜL, Lale ÖCAL, Murat İNANÇ

**Affiliations:** Division of Rheumatology, Department of Internal Medicine, İstanbul Faculty of Medicine, İstanbul University, İstanbul, Turkiye

**Keywords:** Systemic sclerosis, rituximab, disease related outcomes

## Abstract

**Background/aim:**

B-cell depletion with rituximab (RTX) is widely used as a rescue therapy in patients with systemic sclerosis (SSc). The aim herein was to analyze the progress of disease-related outcomes after RTX therapy in severe SSc patients.

**Materials and methods:**

Included in this study were 27 SSc patients who were followed-up between 2012 and 2020 and received at least 1 cycle of RTX for active disease, despite receiving standard immunosuppressives (ISs). In addition to the European Scleroderma Study Group and European Scleroderma Trials and Research Group activity scores, Medsger’s severity, and the recently developed Scleroderma Clinical Trials Consortium Damage Index values were evaluated initially and at 1 year after the first infusion. The progress of individual organ damage was also assessed at the end of the follow-up period (at least 6 months after the last infusion) using the data extracted from the medical records.

**Results:**

Disease activity and severity-improved and disease-related overall damage worsened after the first year of RTX therapy (p < 0.001, p = 0.008, and p = 0.005). Some of the disease-related organ damage had improved at the end of the follow-up period, indicating its reversibility. Overall damage scores ≥11 after the first year of RTX therapy were found to be associated with mortality (p = 0.035).

**Conclusion:**

RTX contributed to reducing the activity and severity in SSc patients with severe disease, nonetheless the efficacy related to the damage was limited. High damage scores in the first year were found to be associated with mortality. Spontaneous progress of manifestations requiring a longer period to improve and irregular consecutive RTX courses might lead to difficulties in differentiation between activity and damage.

## 1. Introduction

Systemic sclerosis (SSc) is a heterogeneous disease characterized by autoimmune inflammation, microangiopathy, and fibrosis of multiple organs. The predominance of one or more of these 3 features mainly identifies the clinical and pathological phenotype of SSc [1]. The pathways leading to tissue damage, inflammation, and fibrosis are of interest to reveal potential therapeutical targets. Previously, therapies have been implemented to reduce inflammation and/or cell proliferation using nonspecific immunosuppression, while new strategies specifically target fibrosis-linked innate and adaptive immune responses [2].

Increasing evidence demonstrating aberrant activation of B cells in SSc fibrosis, B cell depletion with rituximab (RTX), an anti-CD20 antibody, has been studied and favorable effects on skin and pulmonary fibrosis in SSc patients have been displayed [3–8]. Aside from pathogenic antibody production leading to type 1 interferon pathway stimulation, B cells have critical roles including cytokine production, antigen presentation, macrophage differentiation, and altered tissue formation [9]. All of these actions point to B cells as a candidate treatment target for SSc.

In parallel with recent advances related to potential targeted therapies, there has been great effort to develop and validate outcome measures in SSc, including heterogeneous clinical and molecular phenotypes. In addition to the indices of disease activity and severity that have been used to assess SSc patients [10–12], the recently developed Scleroderma Clinical Trials Consortium Damage Index (SCTC-DI) can be useful as an outcome measure [13]. Clinical RTX studies on SSc have commonly focused on improvements in pulmonary and skin manifestations as primary outcomes [14]. In such a heterogeneous autoimmune disease like SSc, when only an organ-based course is evaluated, the general morbidity of the disease may not be monitored adequately. Thus, in addition to individual manifestations, it was aimed herein to analyze the progress of overall activity, severity, and damage after RTX therapy in a well-defined SSc cohort with a long-term follow-up.

## 2. Materials and methods

### 2.1. Patients

This retrospective study included 27 patients who fulfilled European League Against Rheumatism/American College of Rheumatology (2013) classification criteria for SSc and had received at least 1 cycle of RTX due to active disease, despite receiving standard immunosuppressives (ISs). The data were extracted from medical records dated January 1st, 2012, to February 28th, 2020, recorded into a predefined protocol, and analyzed. RTX courses were repeated at 6–12 monthly intervals using off-label approval if disease activity was evident.

### 2.2. Outcomes

Demographics, cumulative organ involvement, treatment, and laboratory data including the acute phase response, blood count, biochemistry, autoantibody profile, and echocardiography (ECHO) were recorded. Pulmonary fibrosis progression was detected using high-resolution computed tomography of the chest and pulmonary function tests together. Pulmonary hypertension was confirmed by right heart catheterization. Gastrointestinal (GI) disease was clinically evaluated, and imaging methods were used if required. Clinically suspicious myositis was confirmed by creatine kinase testing and/or electromyography. Disease activity, severity, and damage outcomes were evaluated before the first infusion and after the first year of RTX therapy. Disease-related organ damage was reevaluated at least 6 months after the last RTX infusion. Disease activity was assessed using the European Scleroderma Study Group (EScSG) Index (different scores of 10 parameters: the presence of a modified Rodnan’s skin score (mRSS) >14, sclerodema, digital necrosis, arthritis, diffusing capacity of the lungs for carbon monoxide (DLCO) <80%, erythrocyte sedimentation rate >30 mm/h, or hypocomplementemia, deterioration of the skin, and vascular or heart/lung with respect to the previous month; the maximum possible total score is 10 points) (defined as an active disease if the total score is ≥3) [10], European Scleroderma Trials and Research Group (EUSTAR) Index (different scores of 6 parameters; presence of a mRSS >14, digital ulcers, tendon friction rubs, C-reactive protein (CRP) >1 mg/dL, DLCO <80%, and deterioration of the skin with respect to the previous month; the maximum possible total score is 10 points) (defined as an active disease if the total score is ≥2.5) [11], and disease severity using Medsger’s Severity Index (score of 0 to 4 for 9 different types of organ involvement; general, peripheral vascular involvement, skin, joint/tendon, myopathy, GI, pulmonary, heart, and renal) [12]. Disease-related damage was assessed using the 23-item multiorgan weighted SCTC-DI (6 different organs/systems; scores are additive with a maximum possible total score of 55). The total scores were also categorized into risk groups, as low (<5), moderate (6–12), and high (≥13) damage [13].

### 2.3. Statistical analysis

Data analysis was performed using IBM SPSS Statistics for Windows 21.0 (IBM Corp., Armonk, NY, USA). Results were expressed as numbers with frequency for the categorical variables and as the mean ± standard deviation (SD), or median and range for the continuous variables. The chi-squared and Fisher’s exact tests were used to compare the prevalence of the categorical variables (baseline characteristics, active disease, severe organ involvement, and presence of organ damage). The Mann–Whitney U test was used to compare the continuous variables (demographics, scores of activity, severity, and damage). Logistic regression analysis was used to analyze mortality-related factors. p ≤ 0.05 was considered statistically significant.

## 3. Results

The prevalence of the disease characteristics of the SSc patients is summarized in Table 1. The mean + SD (range) age, and duration of Raynaud’s and non-Raynaud’s symptoms were 48.3 ± 10.6 (30–70), 9.3 ± 5.7 (3–26), and 7.6 ± 4.2 (3–18) years in the 27 SSc patients (25 females, 89%). The main RTX indications were active disease with predominant skin and lung (n = 9, 33%), skin and musculoskeletal (n = 6, 22%), skin (n = 5, 18.5%), lung (n = 3, 11%), myositis (n = 2, 7.4%), cardiac (n = 1, 7.4%), or musculoskeletal and concomitant digital vasculopathy (n = 1, 7.4%) involvement.

The number of RTX courses (2 infusions of 1000 mg, 15 days apart) given were 1 (n = 4), 2 (n = 6), 3 (n = 10), 4 (n = 1), 5 (n = 2), 6 (n = 1), 7 (n = 2), and 8 (n = 1). ISs were continued in 23 (85.2%) patients concomitantly with RTX. The mean duration of non-Raynaud’s symptoms at the initiation of the RTX therapy was 5.8 ± 4.1 (range: 1–16) years. The mean duration of the follow-up period (years) was 28.9 ± 22.2 months (range: 6–84 months) for RTX therapy.

### 3.1. Disease activity and severity

**Figure 1:**
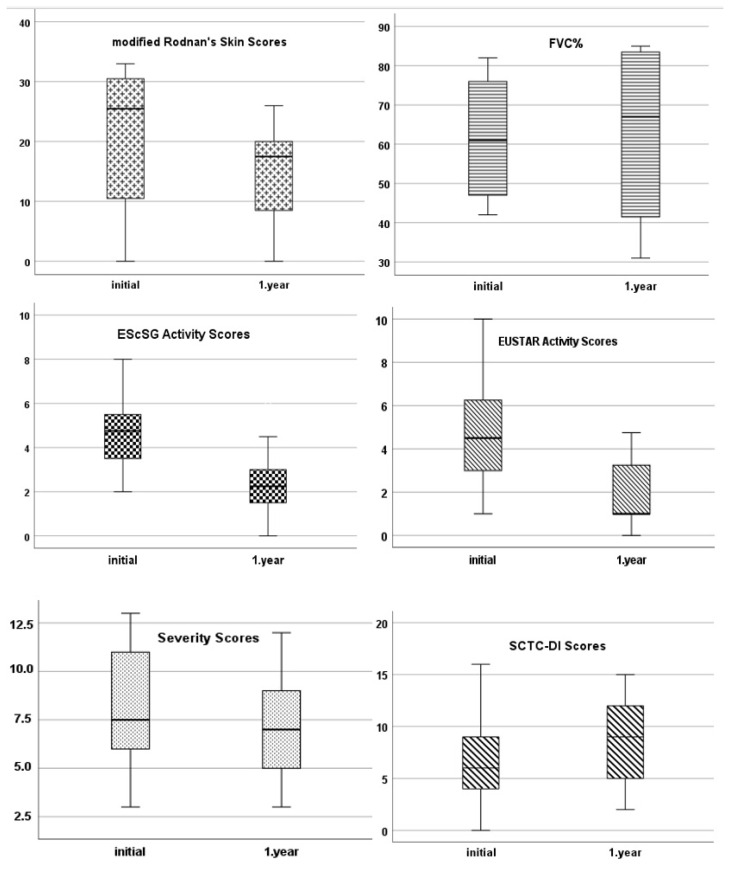
Boxplot graphics of disease-related outcomes after RTX therapy in SSc patients.

After the first year of RTX therapy, the disease activity and total severity scores increased (p < 0.001 for both activity indices and p = 0.008) (Table 2, Figure 1). The frequency of active disease decreased from 92.6% (n = 25) to 30.8% (n = 8) and 81.5% (n = 22) to 42.3% (n = 11) according to the EScSG and EUSTAR indices at the end of the first year. The mRSS increased in patients who received RTX for active skin involvement (p = 0.028). The forced vital capacity (FVC) and DLCO (%) did not significantly change in patients who were treated with RTX for severe and resistant pulmonary fibrosis (Table 2, Figure 1).

### 3.2. Disease related damage

**Figure 2:**
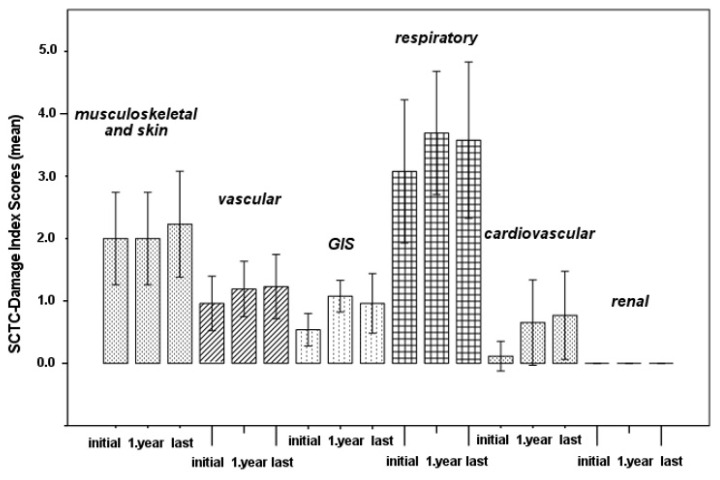
Progress of the disease-related damage scores for different manifestations during RTX therapy in patients with SSc.

After the first year of RTX therapy, the disease-related overall damage worsened (p = 0.005) (Table 2, Figure 1). Before the initiation of RTX, the disease-related damage was evident in 81.5% (SCTC-DI) of the SSc patients despite receiving ISs. The distribution and progress of the damage scores for different manifestations are summarized in Table 2 and Figure 2. After the first year of RTX therapy, the GI damage scores increased (p = 0.010), while the respiratory and cardiovascular damage scores increased nonsignificantly. The vascular, GI, and respiratory damage scores decreased numerically at the end of the follow-up, but there was no significant change (Figure 2).

Regarding the progress of damage, the development of new organ damage or deterioration in the existing damage was observed in 26 manifestations (vascular, GI, respiratory, and cardiovascular) after the first year and in 13 manifestations (musculoskeletal-skin, vascular, GI, and cardiovascular) at the end of the follow-up period. Some of these damaged manifestations improved after RTX therapy; 2 (vascular and respiratory) after the first year and 13 (musculoskeletal-skin, vascular and respiratory, GI) at the end of the follow-up period, indicating the reversibility of the involvement, which was defined as damage. When the total scores were categorized into risk groups as low, moderate, and high, the risk scores increased in 11 (40.7%) patients and decreased in 4 (14.8%) patients after RTX therapy (Table 3).

### 3.3. Safety

Regarding the safety issues, no infusion reactions were detected for RTX. During the treatment period, there were 4 severe infections (pneumonia in 2 patients and infected digital ulcers in 2 patients) and 1 minor infection (an episode of sinusitis). Of the patients, 6 (22.2%) were deceased at the end of the follow-up period (with a mean 114.33 ± 43.06 months of non-Raynaud’s and 43.00 ± 25.29 months of RTX initiation) due to pulmonary hypertension-right heart failure-arrhythmia (n = 3), lung malignancy (n = 2), and opportunistic fungal infection in lungs (n = 1).

### 3.4. Mortality and associated factors

The deceased patients tended to have lower initial DLCO at the initiation of RTX infusion (68.33 ± 14.90 vs. 37.25 ± 26.84, p = 0.058) and were shown to have higher EScSG activity, total damage, and respiratory damage scores after the first year (1.85 ± 1.14 vs. 3.67 ± 1.44, p = 0.011; 7.55 ± 4.05 vs. 12.17 ± 1.47, p = 0.013; and 2.86 ± 2.41 vs. 6.0 ± 0.0, p = 0.012, respectively) and at the end of the follow-up period (2.03 ± 1.16 vs. 3.33 ± 1.21, p = 0.033; 7.38 ± 5.37 vs. 12.17 ± 0.98, p = 0.026; and 2.60 ± 2.68 vs. 6.83 ± 2.04, p = 0.007, respectively). An initial respiratory damage score ≥6 was not significantly associated with mortality (OR: 10, 95% CI: 0.972–102.868, p = 0.053), while a total damage score ≥11 after the first year of RTX was significantly associated with mortality (OR: 12.5, 95% CI: 1.196–130.612, p = 0.035).

## 4. Discussion

Herein, analysis of a single cohort to determine the efficacy of RTX on overall activity, severity, and damage outcomes in patients with severe SSc resistant to ISs was conducted. The data obtained suggests that disease activity and disease severity were improved after RTX therapy. Skin scores were shown to be decreased and the FVC was stabilized in patients who received RTX for resistant skin and pulmonary involvement. Organ damage had already frequently existed in the SSc patients despite receiving ISs and the overall damage continued to worsen during therapy. Regarding the individual manifestations, musculoskeletal and skin were initially the most frequently damaged organs and did not significantly worsen after RTX therapy. GI damage was observed to significantly increase within the first year of therapy, and despite nonsignificant changes, the vascular, respiratory, and cardiovascular systems were the other predominant systems that contributed to overall disease-related damage. After the follow-up period, some of the damaged manifestations, such as GI, vascular, and respiratory, were observed to have recovered, which indicated the limitation in distinguishing between activity and damage. A total damage score ≥11 after the first year of RTX therapy was found to be associated with mortality.

In this cohort, RTX was preferred in 85% of the patients as an add-on treatment, all of whom had resistant SSc with predominantly severe diffuse skin and musculoskeletal involvement. These initial characteristics and leading indications for RTX were compatible with a large multicenter prospective study of SSc patients from the EUSTAR cohort, which included 254 patients who were treated with RTX in daily practice [15]. In addition to severe pulmonary and skin manifestations, severe musculoskeletal involvement that may lead to disability was another important indication for RTX, which was in line with the results herein. Similarly, but less frequently than in the current cohort, more than half of the patients continued ISs concomitantly to RTX in the EUSTAR SSc cohort; skin, joint manifestations, and CRP levels increased; however, lung function was stabilized after RTX therapy. A recently reported study of RTX biosimilar along with 63% concomitant ISs [mycophenolate mofetil (MMF) or methotrexate (MTX)] also revealed improvements in skin, arthritis, CRP, and stabilization in pulmonary functions after 6 months, and the results were similar between the monotherapy and concomitant IS groups [16].

In such a complex and heterogeneous disease, it was thought that the use of composite indices to evaluate the treatment effect of RTX might be a holistic approach, rather than only evaluating the efficacy on specific organs separately. The overall disease activity and severity, and disease-related damage are the outcomes that determine the impact of the disease, morbidity, or well-being on an individual basis, and this required a therapeutical approach. Therefore, the progress of these outcomes after RTX therapy was evaluated. It was shown that disease activity improved significantly in the first year but did not completely decrease to inactivity levels and one-third of the patients were still found to have active disease. The multicenter open-label prospective study of Melsens et al. evaluated the efficacy of RTX (2 courses, 6 months apart) in 17 consecutive SSc patients with early diffuse SSc (within ≤4 years, skin score ≥14) and an EScSG activity score ≥3 [17]. ISs were not used, except for stable doses of MTX and/or steroids (≤10 mg/day). After 24 months of RTX treatment, the skin scores had decreased by 51% (25.5 ± 6.0 to 12.6 ± 5.1 points), and the activity scores had decreased by 63% (4.1 ± 1.7 to 1.5 ± 1.8 p < 0.0001), some patients were still found to have active disease, compatible with the results of the current study. Pulmonary function was stabilized and there was no new organ involvement after RTX therapy, contrary to the results obtained herein.

In a prospective, randomized controlled study by Sircar et al., evaluating the efficacy of RTX vs. cyclophosphamide (CYC) (monthly pulses of 500 mg/m for 2–6 months) in a homogeneous cohort that only included patients with diffuse cutaneous involvement and anti-Scl70 positivity, it was demonstrated that the FVC values increased and mRSS decreased in favor of RTX. The baseline characteristics of their study group, which included younger patients (mean age of 35 years) with early disease (within 3 years) who were not exposed to ISs or biologics may have facilitated the display of the striking efficacy of RTX. In their study, the total severity scores decreased after both RTX and CYC; however, the change was similar between the groups (mean of −3.66 vs. −3.66 points) [3]. In the current study, the total severity scores decreased after RTX therapy.

A metaanalysis of the pooled results of 14 RTX studies, including 597 SSc patients who predominantly had diffuse cutaneous disease, revealed modest effects on skin involvement and the stabilization of pulmonary functions [14]. However, in a randomized placebo-controlled trial of RTX (2 doses of 1000 mg at initiation and a single dose of 1000 mg at 6 months) in 16 patients with early disease, with concomitant ISs, no significant effect was observed in the clinical outcomes, such as skin scores, pulmonary function, and the disability index [18]. Narváez et al. demonstrated that RTX was effective as an add-on to MMF background therapy in patients with worsening SSc-associated interstitial lung disease (ILD) who had no success with ISs (MMF or CYC) and lead to serve pulmonary function [4]. The patients in the current study frequently received RTX as an add-on to MMF or MTX due to severe major organ involvement (pulmonary or cardiac) or resistant musculoskeletal manifestations requiring additional immunosuppression. The effect of concomitant drugs on disease outcomes was not analyzed due to the small sample size.

A recently developed damage index for SSc, namely the SCTC-DI, was intended to identify damage in 6 organs/systems, predicting mortality risk. It was shown herein that initial damage was frequent in SSc patients who required RTX (82%) and the total damage scores increased despite treatment. RTX was found to be effective for some manifestations in the current SSc cohort, reducing the activity and severity, but was insufficient to prevent damage in some patients. In the present cohort, within the first year, most of the SSc patients developed new or worsening organ damage, despite receiving RTX. A moderate to high damage score (≥11) in the first year, despite receiving RTX, was found to be associated with mortality. It was observed that some of the damaged manifestations improved after 2 years of follow-up, indicating that these reversible changes were not compatible with the damage definition. This is an important limitation of SCTC-DI scoring, which requires at least 6 months of existence for all SSc-attributed items. Some parameters, such as peripheral vasculopathy, myositis, resistant gastro-esophageal reflux, or dysphagia, may require a longer period to improve in their course. Moreover, these active SSc patients who needed RTX due to resistant disease, despite receiving ISs, may have had ongoing activity within the first year and clinically relevant responses to RTX that occur later, possibly after repeated courses. Despite the limitations in assessing damage for some manifestations in SSc, it will be useful for predicting high-risk patients and determining tight control strategies for the therapeutic approach. In addition, the progress of the damage demonstrated the unmet need in some SSc-associated manifestations and pointed out that new strategies should be developed.

This study had some limitations. This single-centered study included a small number of patients, and the analyzed data were extracted from medical records. On the other hand, this was a well-defined cohort with long-term follow-up. RTX administration could not be given regularly at 6-month intervals apart due to off-label approval requirement. Efficacy was not evaluated consecutively after all of the RTX infusions. The analysis using objective outcome measures of the overall activity, severity, and damage, and the progress of separate organs/systems in SSc after treatment is of importance. Severe infections and infusion reactions during the treatment period were stated in the study; however, minor infections other than those stated, managed locally in daily practice, and not recorded on medical charts cannot be ruled out.

In conclusion, RTX was prescribed, mostly as an add-on treatment, in SSc patients with progressive disease, despite receiving standard IS treatment, who had predominantly severe diffuse skin, ILD, and musculoskeletal involvement. Disease activity and severity improved after RTX treatment. Disease-related damage worsened during RTX treatment in these patients with frequent initial organ damage. Moderate to high damage scores in the first year were associated with mortality. It should be kept in mind that some manifestations that are improperly considered as damage may be reversible and require more time to recover. There seems to be an unmet need for the treatment of these irreversibly damaged major organs despite receiving advanced treatments. Prospective controlled studies evaluating the overall outcomes in homogenous patient groups with early disease periods will better clarify the efficacy of targeted therapies.

## Figures and Tables

**Table 1:** Prevalence of the characteristics of patients with SSc treated with RTX therapy.

	SSc patients (n = 27)
Clinical characteristics n (%)	
Diffuse cutaneous involvement	22 (81.5)
Digital ulcers	15 (55.6)
Synovitis	12 (44.4)
Flexion contractures	11 (40.7)
Tendon friction rubs	8 (29.6)
Myositis	4 (14.8)
Renal crisis	1 (3.7)
GI involvement	20 (76.9)
Arrhythmia	4 (14.8)
Lung fibrosis	19 (70.4)
Pulmonary hypertension^*^	4 (14.8)
Serology	
ANA	25 (92.6)
Anticentromere	2 (7.4)
Anti-Scl70	18 (66.7)
Previous treatment	
CYC	19 (70.4)
MMF	19 (70.4)
Azathioprine	12 (44.4)
MTX	16 (59.3)
Glucocorticoids	27 (100)
Specific vasodilators	7 (25.9)
Concomitant treatment with RTX	
MMF	17 (62.9)
MTX	6 (22.2)

**Table 2:** Progress of disease-related outcomes during RTX therapy in patients with severe SSc.

Outcome scores mean ± SD, median (min–max)	Pre-RTX initiation	Post-RTX 1 year	p-value
Modified Rodnan’s skin score*	21.7 ± 12.9	15.0 ± 6.3	**0.028**
	25 (14–37)	15 (10–28)	
FVC (%) **	70.7 ± 15.4	74.3 ± 19.4	0.317
	71 (52–86)	85 (52–92)	
DLCO (%) **	51.6 ± 22.9	53.8 ± 11.3	0.655
	52 (21–81)	58 (38–62)	
EScSG activity	4.89 ± 1.82	2.27 ± 1.42	**0.000**
	4.5 (2–9)	2.25 (0–6)	
EUSTAR activity	4.57 ± 2.68	2.62 ± 2.32	**0.000**
	4.5 (1–10)	1.75 (0–9)	
Medsger’s severity (total)	8.00 ± 2.86	7.03 ± 2.38	**0.008**
	7 (3–13)	7 (3–12)	
SCTC damage total	6.29 ± 4.09 6 (0–17)	8.73 ± 4.15 9 (2–15)	**0.005**
Musculoskeletal skin	2.00 ± 1.83	2.00 ± 1.83	0.135
	2 (0–7)	2 (0–7)	
Vascular	1.00 ± 1.07	1.19 ± 1.10	0.097
	0 (0–3)	0 (0–3)	
GI	0.52 ± 0.64	1.08 ± 0.63	**0.010**
	0 (0–2)	1 (0–2)	
Respiratory	3.04 ± 2.78	3.69 ± 2.45	0.819
	2 (0–6)	2 (0–6)	
Cardiovascular	0.11 ± 0.58	0.65 ± 1.70	0.097
	0 (0–3)	0 (0–7)	
Renal	0.11 ± 0.58	0.12 ± 0.59	0.134
	0 (0–3)	0 (0–3)	

EScSG: European scleroderma study group. EUSTAR: European scleroderma trials and research group.

* In patients with active skin.

** In patients with pulmonary fibrosis.

**Table 3:** Prevalence of disease-related organ damage in patients with SSc.

	Initial	1 year	Last
Damage risk groups n (%)			
Low scores (≤5)	11 (40.7)	7 (25.9)	8 (29.6)
Moderate scores (6–12)	14 (51.9)	13 (48.1)	11 (40.7)
High scores (≥13)	2 (11.1)	6 (22.2)	7 (25.9)
Presence of Damage n (%)			
Musculoskeletal-skin	14 (51.9)	-	1 New
		-	3 Deterioration
		-	1 Improvement
Vascular	12 (44.4)	4 New	1 New
		1 Deterioration	3 Deterioration
		1 Improvement	1 Improvement
GI	11 (40.7)	10 New	-
		2 Deterioration	4 Deterioration
		-	7 Improvement
Respiratory	13 (48.1)	6 new	-
		-	3 Deterioration
		1 Improvement	4 Improvement
Cardiovascular	2 (7.4)	3 New	1 New
Renal	1 (3.7)	-	-
